# Identification of miR-16 as an endogenous reference gene for the normalization of urinary exosomal miRNA expression data from CKD patients

**DOI:** 10.1371/journal.pone.0183435

**Published:** 2017-08-31

**Authors:** Tim Lange, Sylvia Stracke, Rainer Rettig, Uwe Lendeckel, Jana Kuhn, Rabea Schlüter, Volkhard Rippe, Karlhans Endlich, Nicole Endlich

**Affiliations:** 1 Department of Anatomy and Cell Biology, University Medicine Greifswald, Greifswald, Germany; 2 Department of Internal Medicine A, Nephrology, University Medicine Greifswald, Greifswald, Germany; 3 Institute of Physiology, University Medicine Greifswald, Karlsburg, Germany; 4 Department of Medical Biochemistry and Molecular Biology, University Medicine Greifswald, Greifswald, Germany; 5 Clinic for Diabetes and Metabolic Diseases, Karlsburg Hospital Dr. Guth GmbH & Co KG, Karlsburg, Germany; 6 Imaging Centre of the Department of Biology, Ernst-Moritz-Arndt University, Greifswald, Germany; 7 Centre for Human Genetics, University of Bremen, Bremen, Germany; Saint Louis University, UNITED STATES

## Abstract

Chronic kidney disease (CKD) is a severe disorder with an increasing incidence worldwide. An early detection may help to prevent its progression and to minimize the risk of cardiovascular diseases as one of the major comorbidities. Recently, extracellular miRNAs like urinary exosomal miRNAs became of great interest as non-invasive biomarkers which can be determined by RT-qPCR. But until now, there is no consensus regarding the normalization of miRNAs isolated from body fluids. The present study analyzed the miRNAs miR-16, miR-92a, miR-21, miR-124a and the small nuclear RNA RNU6B for their applicability as an endogenous reference gene in expression studies of exosomal miRNAs isolated from CKD patients. For this purpose, miRNA expression levels were determined by RT-qPCR after the isolation of urinary exosomes from 33 CKD patients and from 5 healthy controls. Expression data was analyzed with the normalization determination software NormFinder, BestKeeper, GeNorm and DeltaCt. Our results revealed an abundant expression of the four candidate miRNAs in urinary exosomes and no detectable expression of RNU6B. We identified miR-16 as the most stable endogenous reference gene in our data set, making it a suitable endogenous reference gene for miRNA studies of urinary exosomes derived from CKD patients.

## Introduction

With 10% of the population affected in Europe [1], chronic kidney disease (CKD) represents a major public health burden. Unfortunately, there is no causal therapy, terminally leading to renal replacement therapy like hemodialysis or transplantation. Besides inflammatory and autoimmune diseases, CKD mostly arises from diabetes mellitus type II and or arterial hypertension, with glomerulopathies representing the main pathophysiological correlate leading to end-stage renal disease (ESRD) [2]. Effacement or loss of podocytes, a terminally postmitotic cell type and essential part of the glomerular filtration barrier, have been identified as key players in the pathogenesis of glomerulopathies [3]. Since early detection could help to decelerate the progress of CKD and could lower the risk for cardiovascular diseases as secondary diseases, identification of suitable biomarkers plays an essential role in CKD research. As already demonstrated, urinary expression of essential podocyte genes could have been shown to be a more sensitive stress biomarker than standard clinical parameters [4]. miRNAs, small (22 nt) non-coding RNAs regulating the gene expression by their binding to a target mRNA, have recently been discovered as useful clinical markers. They are expressed intracellularly and in all body fluids in a tissue- and developmental stage-specific manner [5]. It was recently shown that miRNAs can be secreted in microvesicles as well as in exosomes [6]. As part of the RNA-induced-silencing-complex (RISC), miRNAs block the translation or induce the degradation of the specific target mRNA [7–9].

Urine plays a prominent role as a non-invasive source of exosomal miRNAs that have previously been identified as biomarkers for several diseases. Currently, little is known about exosomes derived from injured podocytes. Lv and coworkers have identified miR-29c as being significantly down-regulated in CKD patients compared to healthy people. Furthermore, miR-29c expression correlated with estimated glomerular filtration rate (eGFR) and the degree of fibrosis [10]. Moreover, the expression of miR-145 and miR-130 was significantly up-regulated in microalbuminuric patients, whereas the expression of miR-155 and miR-424 was down-regulated. These results were confirmed in animal experiments [11].

To guarantee a high specificity and reproducibility, independently of the applied RT-qPCR (SYBR or Taqman™ probes), a reliable normalization strategy is essential [12]. Currently, the most common method to standardize the assay is to use an endogenous control for relative expression analyses [13,14]. A suitable reference gene needs to be stably and abundantly expressed, which is often not fulfilled.

In the present study, we investigated the suitability of the four different miRNAs miR-16, miR-21, miR-92a and miR-124a as reference genes for the analysis of urinary exosomes miRNAs isolated from CKD patients.

## Material and methods

### Samples

The samples were obtained from urine of 33 CKD patients within the scope of the Greifswald Approach to Individualized Medicine (GANI_MED) [15]. All individuals signed a written informed consent to participate in the study. The study follows the ethical rules of the declaration of Helsinki. Urine provided by five healthy employees of the Department of Anatomy and Cell Biology of the University Medicine Greifswald.

### Urine processing

Morning urine of the patients was processed within 4 hours after voiding, since no degradation was observed within this time span in pre-trials. Approximately 50–100 ml urine were transferred to Falcon tubes and centrifuged for 3 min at 2000 x g to remove any cells or cell debris. The supernatant was transferred to fresh Falcon tubes and stored at -80°C. For further procession, samples were thawed overnight at 4°C. Then the samples were centrifuged again at 2500 x g for 15 min to remove any residual cell debris. 10 ml of the supernatant were transferred to 15 ml tubes for exosome preparation.

### Electron microscopy

For transmission electron microscopy (TEM), 10 mL of cell- and debris-free urine were treated with ExoQuick-TC (System Biosciences, Mountain View, CA, USA) for exosome isolation purposes according to the manufacturer`s instructions with minor modifications. To 10 ml urine, 3.3 mL ExoQuick-TC were added and stored overnight at 4°C. Then, samples were centrifuged at 10,000 x g for 60 min and the supernatant was discarded. The pellet was prepared for TEM according to Théry and coworkers [16] with minor modifications in step 6: 1% uranyl acetate was used to contrast the samples instead of uranyl-oxalate.

### Exosome preparation and miRNA isolation

Exosome preparation was performed with the Urine Exosome Purification and RNA Isolation Midi Kit (Norgen, Thorold, ON, Canada) according to manufacturer’s instructions with minor modifications. The centrifugation steps were performed as described in the urine processing part, to ensure cell- and debris-free urine. After elution of the exosome fraction, the exosome solution was treated with 0.0125 U/μl of the RNase cocktail RiboShredder (Epicentre, Madison, WI, USA) for 30 min at 37°C, to eliminate cell-free non-exosomal RNA. After RNase treatment, the exosome solution was stored on ice and Lysis Buffer A plus Lysis Additive B were added immediately. RNA was eluted in 75 μl Elution buffer. The miRNA concentrations were determined with the Qubit miRNA-Assay and the Qubit 2.0 (Thermo Fisher Scientific, Waltham, MA, USA) according to manufacturer`s instructions using 15 μl sample volume.

### Exosome characterization

Three different exosome fractions from two samples obtained with the Urine Exosome Purification and RNA Isolation Midi Kit (Norgen, Canada) were taken for exosome characterization by Western blot. The first fraction was obtained from 400 μl supernatant of the resuspended and centrifuged Slurry Buffer pellet. The second fraction was the first filtrate containing the cleaned up exosomes. The third fraction was the RiboShredder treated exosome suspension. To each 400 μl fraction, 100 μl of ExoQuick-TC were added, mixed by inversion and incubated at 4°C for 4 h, to precipitate exosomes. Then the samples were centrifuged at 14,000 x g for 30 min and the supernatants were discarded. Exosomes from cell culture supernatants of human podocytes (kindly provided by Dr. Pavenstädt, Münster, Germany) and HEK293 cells served as positive controls for Western blot. Exosomes were isolated with ExoQuick-TC following the manufacturer’s instructions. The pellets from urine samples and cell culture samples were resuspended in Pierce IP Lysis Buffer (500 mM, Thermo Fisher Scientific) with 1 x Halt Proteinase Inhibitor Cocktail (Thermo Fisher Scientific) and 1 x EDTA (0.1%, Thermo Fisher Scientific). Then, the suspension was shaken for 20 min at 1,400 rpm at 4°C in a Thermo Mixer Comfort (Eppendorf, Hamburg, Germany). Afterwards, the samples were centrifuged for 20 min at 14,000 x g at 4°C and the supernatants were transferred to a fresh tube and stored at -20°C.

### Western blot analyses

The protein samples were measured with the Piece BCA Protein Assay Kit (Thermo Fisher Scientific) according to the manufacturer’s instructions. Then, the samples were adjusted to 20 μg/lane (TSG101) and to 10 μg/lane (CD9 and CD63), mixed with 6 x sample buffer (0.35 M Tris pH 6.8, 0.35 M SDS, 30% v/v Glycerol, 0.175 mM bromophenol blue) and boiled at 95°C for 5 min. The protein samples were separated on a 4–20% gradient Mini-Protean TGX Gel Stain-free (Bio-Rad, Hercules, CA, USA). The separated proteins were blotted on nitrocellulose membranes using the Trans-Blot Turbo RTA Transfer Kit (Bio-Rad) and the Trans-Blot Turbo Transfer System (Bio-Rad) at 2.5 A/25 V for 5 min. Membranes were washed in 1x TBS+T wash buffer (50 mM Tris, 150 mM NaCl, 10 mM CaCl_2_, 1 mM MgCl_2_ supplemented with Tween-20 0.1%; AppliChem) and blocked in wash buffer supplemented with 5% milk powder (blocking solution) for 1 h at room temperature. Primary antibodies were diluted in blocking solution and incubated with the membranes overnight. After washing 3 x 5 min with wash buffer, the membranes were incubated with secondary antibodies for 45 min, washed again 4 x 5 min, developed with the ECL Prime Western Blotting Detection Reagent (Amersham) and visualized on X-ray films (Amersham, Hyperfilm ECL) by using Carestream Kodak autoradiography GBX developer/fixer solutions. For normalization and usage of alternative antibodies on the same blot, blots were stripped. Antibodies were used at the following final concentrations: anti-TSG101 (Sigma-Aldrich, 1:1000), anti-CD9 (Invitrogen, 1:2000) and anti-CD63 (Invitrogen; 1:8000).

### Dynamic light scattering analysis

For dynamic light scattering analysis, 10 ml of three different cell- and debris-free, representative urine samples were treated with ExoQuick-TC as already described. To 10 ml urine, 3.3 ml ExoQuick-TC was added and stored overnight at 4°C. Samples were centrifuged at 10,000 x g for 60 min and the supernatant was discarded. The pellets were resuspended in 1.5 ml ice cold PBS. Then samples were vacuum-degassed for 20 min at RT in a ThermoVac (GE, Little Chalfont, UK). Afterwards 200 μl of each sample were transferred to a 1 cm path lenght cuvette (Brandt, Wertheim, Germany). Each sample was measured 5 times with a Zetasizer Nano SZ (Malvern, Herrenberg, Germany) at 25°C with a refraction index of 1.36 and an absorption of 0.001 with standard diluent parameters as referred to H_2_O. Each measurement consisted of 20 runs ā 10 sec. Data was analyzed with the Zetasizer Software 7.11 and displayed as size/intensity plots.

### Selection of the normalization candidates

Candidates were selected by evidential expression in urinary exosomes and/or evidential use as normalizers in previous circulating miRNA studies. RNU6B was also chosen since it is broadly used as normalizer for intracellular miRNA studies as well as for circulating miRNA studies (Table 1).

**Table 1:** Endogenous normalization candidates.

Candidate	Type	Accession# MiRBase / NCBI	Evidence
hsa-miR-16-5p	miRNA	MIMAT0000069	[17,18]
hsa-miR-21-5p	miRNA	MIMAT0000076	[17,18]
hsa-miR-92a-3p	miRNA	MIMAT0000092	[17–19]
hsa-miR-124a-3p	miRNA	MIMAT0000422	[18]
RNU6B	snRNA	NR_002752	

### Reverse transcription

cDNA was synthesized starting from 1 ng miRNA using Taqman™ miRNA Assays and the Taqman™ miRNA Reverse Transcription Kit (Thermo Fisher Scientific). The following Taqman™ miRNA Assays for candidate normalizers were used in this study: Hsa-miR-16-5p: ID #000391; Hsa-miR-21-5p: ID #000397; Hsa-miR-92a-3p: ID #000431; Hsa-miR-124a-3p: ID #001182. We also tested a broadly used Taqman™ miRNA Assay for miRNA normalization: RNU6B: ID #001219. In case of less than 1 ng RNA, 5 μL of RNA solution were used. The setup for RT-reactions was performed after the manufacturer`s instructions. One ng placenta RNA was used as positive control and inter-run-calibrator and was co-synthesized with every cDNA synthesis run. Negative controls included no template and no reverse transcriptase controls.

### RT-qPCR

The qPCR was performed with the Taqman™ miRNA Assays described above and the Taqman™ Universal Master Mix II, no UNG (Thermo Fisher Scientific) following the manufacturer’s instructions. The reaction volumes contained 1.33 μl undiluted cDNA solution and 18.67 μl Master Mix. The qPCR was performed on the Light Cycler^®^ Nano (Roche Applied Biosystem, Mannheim, Germany) with the following cycler scheme: 10 min at 95°C followed by 45 cycles of 15 sec at 95°C and 60 sec at 60°C. All samples were run in triplicate and placenta cDNA was used as inter-run-calibrator. Negative controls included the ones from cDNA synthesis and an extra no template control for the qPCR reaction. Standard curves with standard cDNA samples were used for efficiency determination of every single Taqman™ miRNA Assay. C_t_-values were calculated by the Light Cycler^®^ Nano SW 1.1 software (Roche Applied Biosystem) with automatically set thresholds and baselines. Raw C_t-_values ≥38 were excluded from analysis. All C_t_-values were normalized against starting RNA amount and were inter-run calibrator corrected.

### Normalization analysis

Normalization analysis was performed using different online available normalization tools. The first one is NormFinder v0.953 (http://moma.dk/NormFinder-software), which works with an algorithm for linear data to determine the most stable normalization candidate gene [20]. The tool calculates a stability value (SV) for every single candidate. The lower the stability value, the more stable is the expression of the corresponding candidate gene. The data output comes with the stability values for all candidates together with standard deviations (SD) and announces the most stable candidate gene. Splitting data into study groups, results in additional intra- and intergroup variation and determination of the best endogenous normalizer combination. The second tool used in this study is the online-based tool RefFinder [21] (http://fulxie.0fees.us/). It comprises four different commonly used normalization tools, namely BestKeeper [22], comparative DeltaCt [23,24], NormFinder [20] and GeNorm [25], working with different algorithms to evaluate the most stably expressed gene or gene pair of a specific sample set.

### Statistical analyses

All statistical analyses were performed with IBM SPSS Statistics 22.0 (SPSS Inc., Chicago, IL, USA). Data was tested for normality by Kolmogorov-Smirnov test. Differences between two groups were tested with Mann-Whitney-*U*-test. The two-tailed tests with p-values ≤ 0.05 were considered statistically significant.

## Results

### Characterization of urinary exosomes

**Fig 1:**
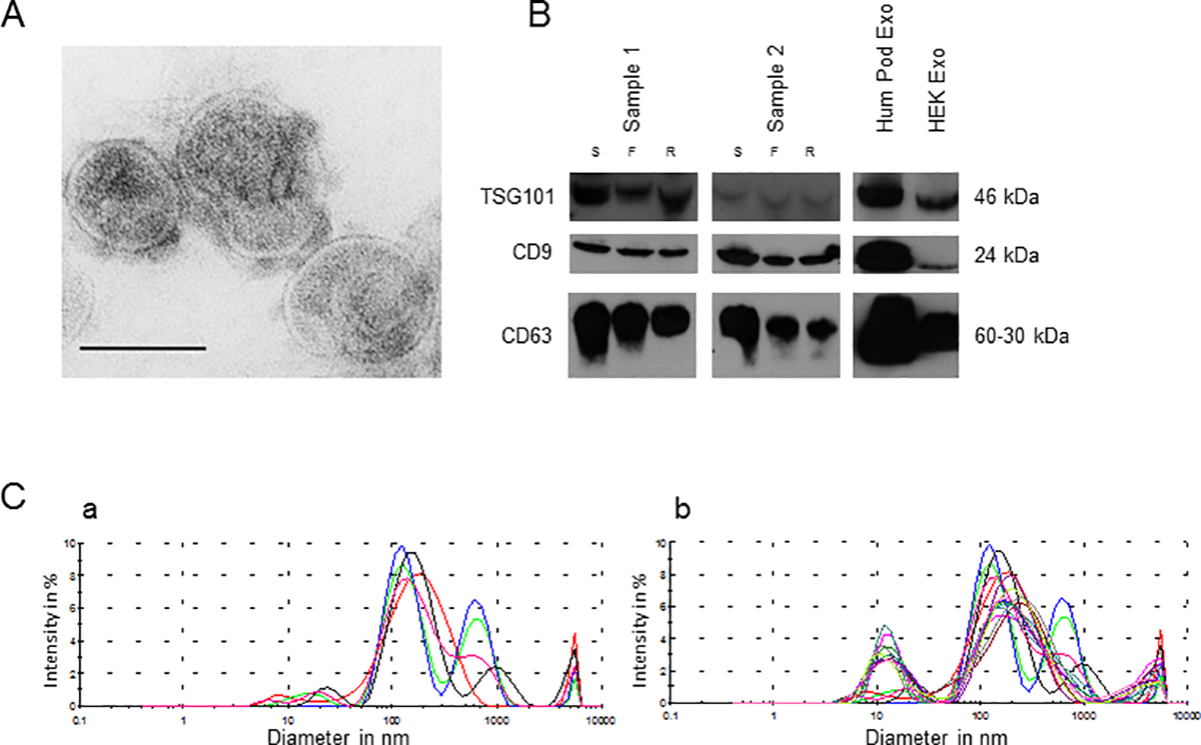
Characterization of urinary exosomes. Urinary extracellular vesicles were isolated with ExoQuick-TC and prepared for TEM. Vesicles showed an exosome-like shape and size. Scale bar = 100 nm (A). Exosomes of two samples from Norgen urine exosome preparation kit were precipited with ExoQuick-TC and analyzed by Western blot for exosomal markers TSG101, CD9 and CD63. Protein extracts display the presence of exosomal marker proteins over the whole preparation procedure with a slight decrease of band intensities. S = slurry pellet, F = filtrated exosome suspension and R = filtrated exosome suspension after RiboShredder treatment (B). Zetasizer analysis of urinary exosome samples (C). The results of the 5 measurements of the urine sample displayed in TEM (A) are shown (C, a). Beside this the results of the 5 measurements per sample of three urinary exosome samples are shown (C, b). The figures show vesicles with typical exosomal size and some bigger structures that might be due to clumping artefacts.

For characterization of exosomes in the urine supernatants, exosomes were isolated from 10 ml urine of 3 CKD patients, using ExoQuick-TC and were prepared for electron microscopy. Fig 1A shows the results obtained from TEM, revealing vesicles with a size of 20–80 nm and a cup-like shape as typical characteristics of exosomes. Furthermore, a dynamic light scattering analysis revealed two main peaks for the sample displaying the sample shown in TEM (Fig 1C a). The biggest peak shows vesicles with a size ranging from approximately 45 nm to 300 nm with its maximum at approximately 130 nm. The second smaller peak has its maximum at 600 nm which is caused by vesicle clumping. The analysis of three different representative urinary exosomal samples showed a relatively homogeneous size distribution throughout the different samples with a similar peak situation as described for the first sample alone (Fig 1C b). Additionally, we performed our exosome preparation protocol as described above on urine samples from two individuals. To confirm that the vesicles obtained were in fact exosomes, protein extracts were analyzed by Western blot for the presence of exosome markers TSG101, CD9 and CD63 (Fig 1B). The Western blot showed specific signals for all three exosome markers in all three preparation fractions with decreasing band intensity of CD63 and CD9 from Slurry Buffer pellet, over exosome filtrate to the RiboShredder-treated fraction. Lysates of human podocytes and HEK293 exosomes served as positive controls.

### Patient characteristics

The patient characteristics are shown in Table 2. In total, 38 individuals were included in this study with a mean age of 62 (±19) years. Eighteen participants were female. The study group included four diabetic patients that were all in the CKD group. The CKD group had a mean age of 66 (±16) years. Fifteen members of this group were female. The healthy control group had a mean age of 40 (±15) years and included three females.

**Table 2:** Patient characteristics. Patient characteristics are displayed as total patients, CKD-patients and the normal group with parameters: mean age in years, SD = Standard deviations; sex in amount patients, percentage and diabetes in amount patients, percentage.

Characteristics	Total	CKD patients	Healthy controls
Age; mean (SD)	62 (±19)	66 (±16)	40 (±15)
Sex; n female/male (%)	18 (47.4)/20 (52.6)	15 (45.5)/18 (54.5)	3 (60.0)/2 (40.0)
Diabetes; n (%)	4 (10.5)	4 (12.1)	0 (0.0)

Patient characteristics are displayed as total patients, CKD-patients and the normal group with parameters: mean age in years, SD = Standard deviations; sex in amount patients, percentage and diabetes in amount patients, percentage

### Expression of mature miR-16, miR-21, miR-92a and miR-124a

**Fig 2:**
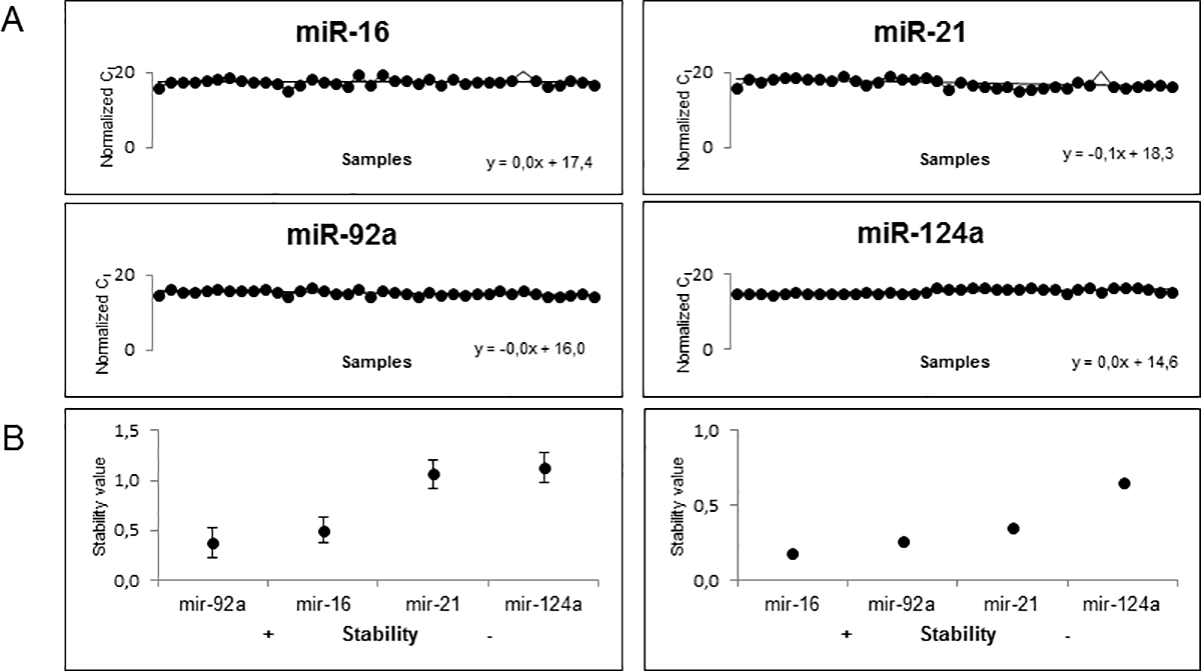
Expression distribution and stability of normalization candidate mature miRNAs. All four candidate miRNAs are abundantly expressed in urinary exosomes of CKD patients and the normal group. Exosomal RNA was isolated with the Norgen urinary exosome preparation and RNA isolation kit. Expression data was generated by qRT-PCR using Taqman® miRNA Assays. The raw Ct-values were normalized against RNA input and an inter-run calibrator (A). MiR-92a is the most stable candidate normalizer within the data set, as revealed by NormFinder software. The best combination is miR-92a/16 with miR-16 as the most stable one, when input data is split into CKD patients and normal group. Error bars = SD (B).

Normalized expression of all four candidates (miR-16, miR-21, miR-92a and miR-124a) showed a relatively high homogeneity over all 38 samples (S1 Table), since the slopes of the trend lines were zero or, in the case of miR-21, close to zero (Fig 2A). These results also showed an abundant expression of the four mature miRNAs in urinary exosomes with median normalized C_t_-values of 17.6, 17.1, 15.3 and 15.1 for miR-16, miR-21, miR-92a and miR-124a, respectively. For RNU6B we could not detect any expression, indicated by raw C_t_-values ≥ 38 (data not shown).

### Stability analysis

#### NormFinder

For stability analysis the whole data set of input RNA and inter-run calibrator-normalized C_t_-values was analyzed with the NormFinder software ignoring classification. As shown in Fig 2B, miR-92a showed the highest stability (SV = 0.38±0.14) followed by miR-16 (SV = 0.51±0.13), miR-21 (SV = 1.07±0.14) and miR-124a (SV = 1.13±0.15). From these results the NormFinder software identified miR-92a as the best normalizer. In a second analysis, data was split into groups comprised of CKD patients and healthy individuals. The results showed that miR-16 appears to be the most stable candidate (SV = 0.18) followed by miR-92a (SV = 0.27), miR-21 (SV = 0.35) and miR-124a (SV = 0.66) (Fig 2B). The best combination of two genes was the combination of miR-16 and miR-92a with a SV of 0.25.

#### RefFinder

**Fig 3:**
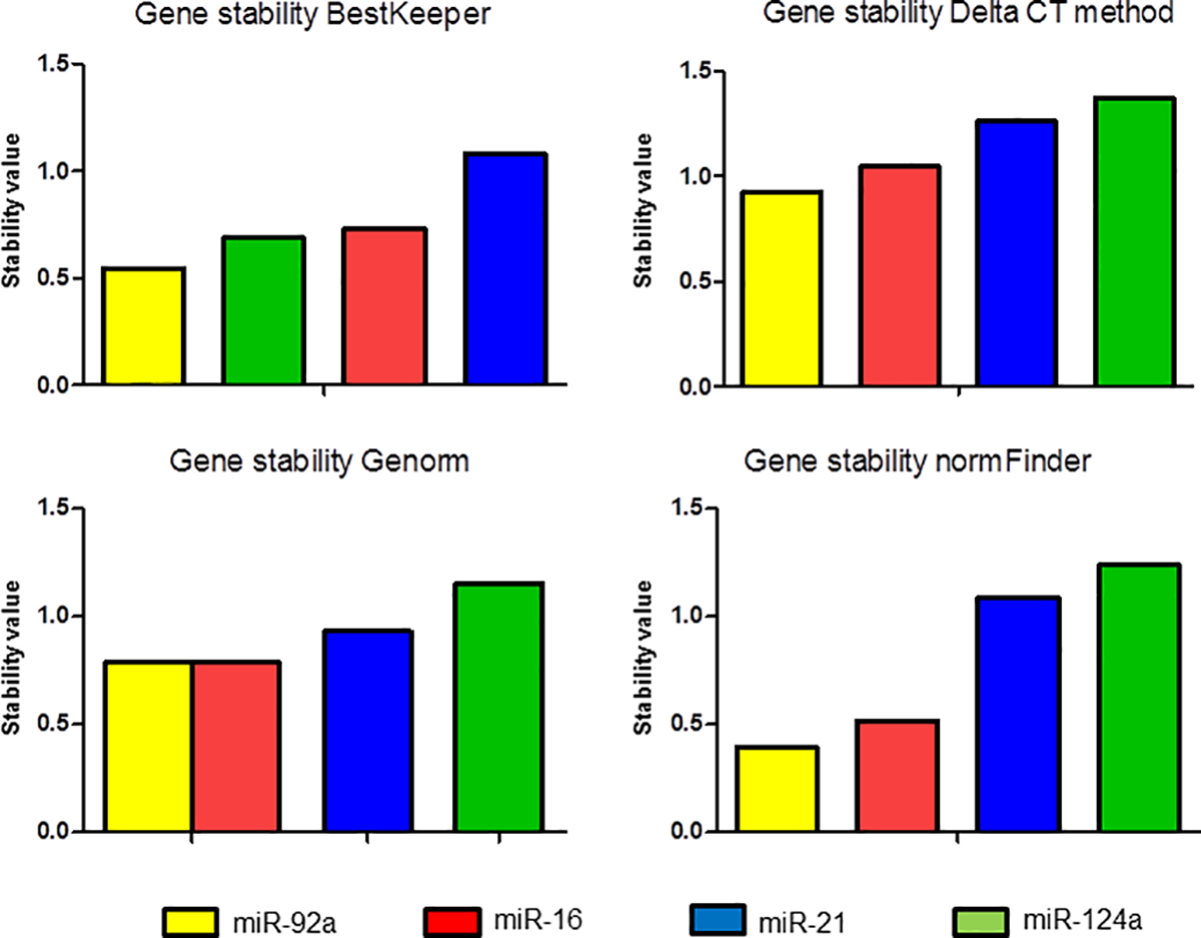
Stability of normalization candidate mature miRNAs determined with different algorithms. Stability values were calculated by the online available tools BestKeeper, DeltaCt, Genorm and NormFinder. The lower the stability value, the higher the stability. Each tool reveals miR-92a as the most stable one or the combination of miR-92a/miR-16. miR-92a = yellow, miR-16 = blue, miR-21 = purple and miR-124a = green.

Additionally, the data set was analyzed with the online tool RefFinder. This tool combines the normalization determination algorithms GeNorm, BestKeeper, DeltaCt and NormFinder. Stability analysis with BestKeeper identified miR-92a as the most stable normalization candidate in the presented data set. miR-92a was followed by miR-124a as the second stable candidate, which in turn was followed by miR-21 and miR-16 (Fig 3). Looking at significant correlations of the potential normalizers with the BestKeeper index, the only candidate that showed a non-significant correlation was miR-124a (data not shown). The comparative DeltaCt method showed the same results as the NormFinder software or the GeNorm analysis that stated miR-16/92a as the most stable gene-pair (Fig 3). The NormFinder results obtained from the software were also confirmed by the online version included in RefFinder (Fig 3).

### Expression differences between CKD patients and healthy controls

**Fig 4:**
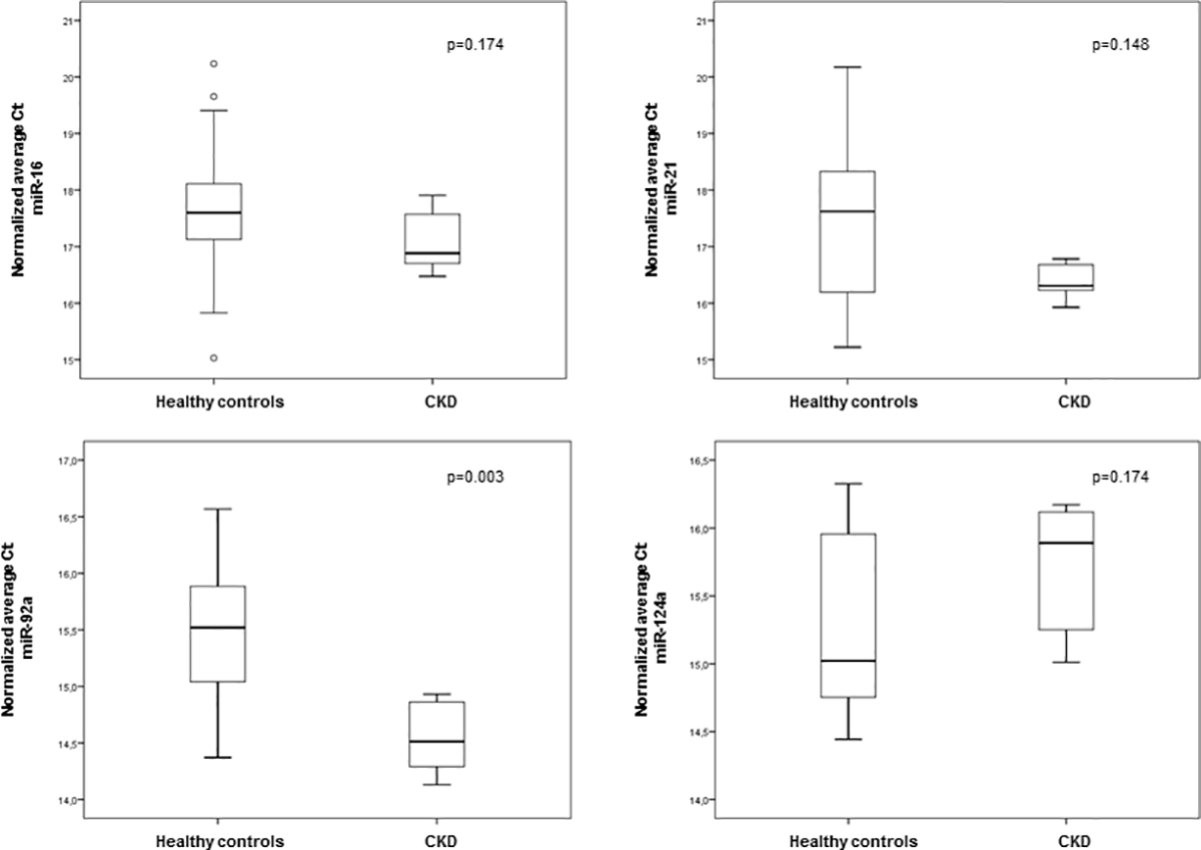
Expression differences of the normalization candidates between healthy controls and chronic kidney disease (CKD) patients. Box-Whisker Plot of the RNA input- and inter-run calibrator-normalized expression values determined by Taqman® qRT-PCR. The midline represents the median and the box borders represent the inter-quantile range. Percentiles are displayed by whiskers.

Since it is essential for an endogenous reference gene to be stably expressed in both, diseased and healthy states, we analyzed our data set for differences in mean C_t_-values between both groups. Fig 4 shows no significant differences between healthy controls and CKD patients in the mean expression of miR-16, miR-124a and miR-21. The only significant difference between these two groups was observed with respect to the expression of miR-92a (Fig 4).

## Discussion

In this study we analyzed miR-16, miR-21, mir-92a and miR-124a as potential reference miRNA genes in urinary exosomes of 33 CKD patients and five healthy controls. Since ultracentrifugation is not very applicable when it comes to high sample throughput due to its low availability and the high time consumption, we used a low-speed centrifugation kit approach which is established for the isolation of exosomes. Furthermore, it has been shown that low-speed centrifugation approaches are superior over ultracentrifugation in terms of total exosome isolation and especially in RNA recovery [26,27]. The presence and characterization of exosomes were confirmed by TEM and Western blot. Transmission electron micrographs revealed extracellular vesicles with typical exosomal characteristics in shape and size [28]. The relatively small vesicle size in urine samples has been described previously [29–31]. This might be due to shrinking processes during TEM preparation procedure, as reported previously [32,33]. Beside TEM we analyzed the size of the exosomes with dynamic light scattering. By using this method we found that the isolated vesicles showed the size typical for exosomes [34] beside some bigger structures that might be due to clumping, which we have also identified by TEM. Since extracellular vesicle cargo is protected against RNase treatment [6,35–37], we included an additional RNase treatment step in our isolation procedure, to eliminate contamination with non-exosomal free-miRNAs. Furthermore, we were able to detect strong signals for the typical exosome markers CD9, CD63 and TSG101 [34] throughout the whole preparation procedure, by Western blots. The TSG101 intensity varied between both samples. It is known that the proportions of exosomal marker proteins can vary between different samples. Royo and co-workers could also observe different TSG101 levels in different samples from prostate adenoma patients with relatively equal CD63 protein levels, considering different exosomal protein loading as a possible reason [38]. The stability of the purified exosomes was also confirmed by the consistent measurement of the described miRNAs.

To limit variations due to different urine concentrations, we used timed urine samples [39,40]. Although three different normalization strategies for miRNA RT-qPCR experiments are available, there is no consensus over universally applicable endogenous controls. One strategy is the normalization by global mean miRNA expression [41]. However, this method requires a remarkable amount of miRNAs per sample and is expensive and time consuming. Another method normalizes the amount of miRNA against a spike-in control like a synthesized miRNA from *Caenorhabditis elegans* such as ce-miR-37. This normalization may consider the experimental influences on the samples, but it does not consider the endogenous state of the overall miRNA expression [42–44]. The most utilized method is the endogenous control method that determines the relative expression of the target gene using abundant and stably expressed endogenous miRNAs [13,14,23]. One of the most frequently used endogenous reference genes for circulating and exosomal miRNA normalization is RNU6B. RNU6B is a small noncoding RNA, but not a miRNA, which is exclusively expressed in nuclei and should not be detectable in isolated exosomes or in fractions of circulating miRNA. Nevertheless, severals studies [10,45–47] recommend RNU6B as a reference gene for quantification of miRNA in body fluids. In the present study, RNU6B was not detectable suggesting that our exosome fraction was pure and did not show any cellular contamination. These results are in agreement with those obtained by Solayman et al. and Sole et al. [17,46], who proposed to use RNU6B as a quality indicator for cellular contaminations and cellular degradation processes. It was reported that miR-92a or the combination of miR-92a with miR-16 are the most reliable candidates for normalization, followed by miR-16 alone [17,48]. In agreement with these reports, the stability analysis of our data with the NormFinder software identified miR-92a as the most stable miRNA followed by miR-16. Using the BestKeeper software, we also found miRNA-92 as the most reliable candidate. The second candidate of the BestKeeper analysis was miR-124a, directly followed by miRNA-16. However, the BestKeeper software did not consider the non-significant correlation of miRNA-124a with the BestKeeper value. Therefore, our analysis revealed that miR-92a and miR-16 are the most stably expressed normalization candidates, which is in agreement with several miRNA expression studies [17,48]. The disadvantage of miR-92a as a normalizer is that it cannot be used as an endogenous reference gene since we found a significant difference in the expression of miR-92a in the control group compared to the CKD group.

Since this is not the case for the second stable candidate of our data analyses, the miR-16, which is already used as an endogenous reference gene in several cancer studies [49–52] and also in studies with hypertensive patients [17], we conclude that miR-16 is the best normalizer for miRNAs of urinary exosomes isolated from CKD patients within the tested candidates.

## Supporting information

S1 Table S1 Table shows the raw miRNA expression data normalized against RNA input and the inter-run calibrator of CKD patients (numbers) and healthy control individuals (N-number). (PDF)

## References

[1] Brück K, Stel VS, Gambaro G, Hallan S, Völzke H, Ärnlöv J, et al CKD Prevalence Varies across the European General Population. Journal of the American Society of Nephrology. 2015 doi: 10.1681/ASN.2015050542 26701975 10.1681/ASN.2015050542 PMC4926978

[2] Wiggins R. The spectrum of podocytopathies: A unifying view of glomerular diseases. Kidney International. 2007; 71: 1205–1214. doi: 10.1038/sj.ki.5002222 17410103 10.1038/sj.ki.5002222

[3] Kriz W. Podocyte is the major culprit accounting for the progression of chronic renal disease. Microsc Res Tech. 2002; 57: 189–195. doi: 10.1002/jemt.10072 12012382 10.1002/jemt.10072

[4] Fukuda A, Wickman LT, Venkatareddy MP, Wang SQ, Chowdhury MA, Wiggins JE, et al Urine podocin:nephrin mRNA ratio (PNR) as a podocyte stress biomarker. Nephrol Dial Transplant. 2012; 27: 4079–4087. doi: 10.1093/ndt/gfs313 22863839 10.1093/ndt/gfs313 PMC3494841

[5] Weber JA, Baxter DH, Zhang S, Huang DY, Huang KH, Lee MJ, et al The microRNA spectrum in 12 body fluids. Clin Chem. 2010; 56: 1733–1741. doi: 10.1373/clinchem.2010.147405 20847327 10.1373/clinchem.2010.147405 PMC4846276

[6] Valadi H, Ekström K, Bossios A, Sjöstrand M, Lee JJ, Lötvall JO. Exosome-mediated transfer of mRNAs and microRNAs is a novel mechanism of genetic exchange between cells. Nat Cell Biol. 2007; 9: 654–659. doi: 10.1038/ncb1596 17486113 10.1038/ncb1596

[7] Bartel DP. MicroRNAs: genomics, biogenesis, mechanism, and function. Cell. 2004; 116: 281–297. 14744438 10.1016/s0092-8674(04)00045-5

[8] Guo H, Ingolia NT, Weissman JS, Bartel DP. Mammalian microRNAs predominantly act to decrease target mRNA levels. Nature. 2010; 466: 835–840. doi: 10.1038/nature09267 20703300 10.1038/nature09267 PMC2990499

[9] Siomi H, Siomi MC. Posttranscriptional regulation of microRNA biogenesis in animals. Mol Cell. 2010; 38: 323–332. doi: 10.1016/j.molcel.2010.03.013 20471939 10.1016/j.molcel.2010.03.013

[10] Lv L, Cao Y, Ni H, Xu M, Liu D, Liu H, et al MicroRNA-29c in urinary exosome/microvesicle as a biomarker of renal fibrosis. Am J Physiol Renal Physiol. 2013; 305: F1220–7. doi: 10.1152/ajprenal.00148.2013 23946286 10.1152/ajprenal.00148.2013

[11] Barutta F, Tricarico M, Corbelli A, Annaratone L, Pinach S, Grimaldi S, et al Urinary exosomal microRNAs in incipient diabetic nephropathy. PLoS One. 2013; 8: e73798 doi: 10.1371/journal.pone.0073798 24223694 10.1371/journal.pone.0073798 PMC3817183

[12] Schmittgen TD, Lee EJ, Jiang J, Sarkar A, Yang L, Elton TS, et al Real-time PCR quantification of precursor and mature microRNA. Methods. 2008; 44: 31–38. doi: 10.1016/j.ymeth.2007.09.006 18158130 10.1016/j.ymeth.2007.09.006 PMC2663046

[13] Schwarzenbach H, da Silva AM, Calin G, Pantel K. Data Normalization Strategies for MicroRNA Quantification. Clin Chem. 2015; 61: 1333–1342. doi: 10.1373/clinchem.2015.239459 26408530 10.1373/clinchem.2015.239459 PMC4890630

[14] Meyer SU, Pfaffl MW, Ulbrich SE. Normalization strategies for microRNA profiling experiments: a 'normal' way to a hidden layer of complexity. Biotechnol Lett. 2010; 32: 1777–1788. doi: 10.1007/s10529-010-0380-z 20703800 10.1007/s10529-010-0380-z

[15] Grabe HJ, Assel H, Bahls T, Dorr M, Endlich K, Endlich N, et al Cohort profile: Greifswald approach to individualized medicine (GANI_MED). J Transl Med. 2014; 12: 144 doi: 10.1186/1479-5876-12-144 24886498 10.1186/1479-5876-12-144 PMC4040487

[16] Théry C, Amigorena S, Raposo G, Clayton A. Isolation and characterization of exosomes from cell culture supernatants and biological fluids. Curr Protoc Cell Biol. 2006; Chapter 3: Unit 3.22. doi: 10.1002/0471143030.cb0322s30 18228490 10.1002/0471143030.cb0322s30

[17] Solayman MHM, Langaee T, Patel A, El-Wakeel L, El-Hamamsy M, Badary O, et al Identification of Suitable Endogenous Normalizers for qRT-PCR Analysis of Plasma microRNA Expression in Essential Hypertension. Mol Biotechnol. 2016; 58: 179–187. doi: 10.1007/s12033-015-9912-z 26798072 10.1007/s12033-015-9912-z PMC4758859

[18] Cheng L, Sun X, Scicluna BJ, Coleman BM, Hill AF. Characterization and deep sequencing analysis of exosomal and non-exosomal miRNA in human urine. Kidney International. 2014; 86: 433–444. doi: 10.1038/ki.2013.502 24352158 10.1038/ki.2013.502

[19] Han H, Jo YN, Lee JY, Choi S, Jeong Y, Yun J, et al Identification of suitable reference genes for the relative quantification of microRNAs in pleural effusion. Oncol Lett. 2014; 8: 1889–1895. doi: 10.3892/ol.2014.2404 25202432 10.3892/ol.2014.2404 PMC4156210

[20] Andersen CL, Jensen JL, Ørntoft TF. Normalization of real-time quantitative reverse transcription-PCR data: a model-based variance estimation approach to identify genes suited for normalization, applied to bladder and colon cancer data sets. Cancer Res. 2004; 64: 5245–5250. doi: 10.1158/0008-5472.CAN-04-0496 15289330 10.1158/0008-5472.CAN-04-0496

[21] Xie F, Xiao P, Chen D, Xu L, Zhang B. miRDeepFinder: a miRNA analysis tool for deep sequencing of plant small RNAs. Plant Mol Biol. 2012 doi: 10.1007/s11103-012-9885-2 22290409 10.1007/s11103-012-9885-2

[22] Pfaffl MW, Tichopad A, Prgomet C, Neuvians TP. Determination of stable housekeeping genes, differentially regulated target genes and sample integrity: BestKeeper—Excel-based tool using pair-wise correlations. Biotechnol Lett. 2004; 26: 509–515. 15127793 10.1023/b:bile.0000019559.84305.47

[23] Pfaffl MW. A new mathematical model for relative quantification in real-time RT-PCR. Nucleic Acids Research. 2001; 29: 45e doi: 10.1093/nar/29.9.e45 10.1093/nar/29.9.e45 PMC55695 11328886

[24] Pfaffl MW. AZ of Quantitative PCR, Chapter 3–Quantification strategies in real-time PCR. International University Line (IUL), La Jolla, CA, USA.

[25] Vandesompele J, Preter K de, Pattyn F, Poppe B, van Roy N, Paepe A de, et al Accurate normalization of real-time quantitative RT-PCR data by geometric averaging of multiple internal control genes. Genome Biol. 2002; 3: RESEARCH0034 12184808 10.1186/gb-2002-3-7-research0034 PMC126239

[26] Royo F, Diwan I, Tackett MR, Zuniga P, Sanchez-Mosquera P, Loizaga-Iriarte A, et al Comparative miRNA Analysis of Urine Extracellular Vesicles Isolated through Five Different Methods. Cancers (Basel). 2016; 8 doi: 10.3390/cancers8120112 27973407 10.3390/cancers8120112 PMC5187510

[27] Alvarez ML, Khosroheidari M, Kanchi Ravi R, DiStefano JK. Comparison of protein, microRNA, and mRNA yields using different methods of urinary exosome isolation for the discovery of kidney disease biomarkers. Kidney International. 2012; 82: 1024–1032. doi: 10.1038/ki.2012.256 22785172 10.1038/ki.2012.256

[28] Théry C, Zitvogel L, Amigorena S. Exosomes: composition, biogenesis and function. Nat Rev Immunol. 2002; 2: 569–579. doi: 10.1038/nri855 12154376 10.1038/nri855

[29] Pisitkun T, Shen R, Knepper MA. Identification and proteomic profiling of exosomes in human urine. Proc Natl Acad Sci U S A. 2004; 101: 13368–13373. doi: 10.1073/pnas.0403453101 15326289 10.1073/pnas.0403453101 PMC516573

[30] Perez-Hernandez J, Forner MJ, Pinto C, Chaves FJ, Cortes R, Redon J. Increased Urinary Exosomal MicroRNAs in Patients with Systemic Lupus Erythematosus. PLoS One. 2015; 10: e0138618 doi: 10.1371/journal.pone.0138618 26390437 10.1371/journal.pone.0138618 PMC4577109

[31] Gracia T, Wang X, Su Y, Norgett EE, Williams TL, Moreno P, et al Urinary Exosomes Contain MicroRNAs Capable of Paracrine Modulation of Tubular Transporters in Kidney. Sci Rep. 2017; 7: 40601 doi: 10.1038/srep40601 28094285 10.1038/srep40601 PMC5240140

[32] Dragovic RA, Gardiner C, Brooks AS, Tannetta DS, Ferguson DJP, Hole P, et al Sizing and phenotyping of cellular vesicles using Nanoparticle Tracking Analysis. Nanomedicine. 2011; 7: 780–788. doi: 10.1016/j.nano.2011.04.003 21601655 10.1016/j.nano.2011.04.003 PMC3280380

[33] Whiteside TL. Tumor-Derived Exosomes and Their Role in Cancer Progression. Adv Clin Chem. 2016; 74: 103–141. doi: 10.1016/bs.acc.2015.12.005 27117662 10.1016/bs.acc.2015.12.005 PMC5382933

[34] Février B, Raposo G. Exosomes: endosomal-derived vesicles shipping extracellular messages. Curr Opin Cell Biol. 2004; 16: 415–421. doi: 10.1016/j.ceb.2004.06.003 15261674 10.1016/j.ceb.2004.06.003

[35] Huang X, Yuan T, Tschannen M, Sun Z, Jacob H, Du M, et al Characterization of human plasma-derived exosomal RNAs by deep sequencing. BMC Genomics. 2013; 14: 319 doi: 10.1186/1471-2164-14-319 23663360 10.1186/1471-2164-14-319 PMC3653748

[36] Koga Y, Yasunaga M, Moriya Y, Akasu T, Fujita S, Yamamoto S, et al Exosome can prevent RNase from degrading microRNA in feces. J Gastrointest Oncol. 2011; 2: 215–222. doi: 10.3978/j.issn.2078-6891.2011.015 22811855 10.3978/j.issn.2078-6891.2011.015 PMC3397623

[37] Mall C, Rocke DM, Durbin-Johnson B, Weiss RH. Stability of miRNA in human urine supports its biomarker potential. Biomark Med. 2013; 7: 623–631. doi: 10.2217/bmm.13.44 23905899 10.2217/bmm.13.44 PMC3885156

[38] Royo F, Zuniga-Garcia P, Sanchez-Mosquera P, Egia A, Perez A, Loizaga A, et al Different EV enrichment methods suitable for clinical settings yield different subpopulations of urinary extracellular vesicles from human samples. J Extracell Vesicles. 2016; 5: 29497 doi: 10.3402/jev.v5.29497 26895490 10.3402/jev.v5.29497 PMC4759834

[39] Waikar SS, Sabbisetti VS, Bonventre JV. Normalization of urinary biomarkers to creatinine during changes in glomerular filtration rate. Kidney International. 2010; 78: 486–494. doi: 10.1038/ki.2010.165 20555318 10.1038/ki.2010.165 PMC3025699

[40] Youhe Gao. Urine Proteomics in Kidney Disease Biomarker Discovery; 2014. p. 50.

[41] Mestdagh P, van Vlierberghe P, Weer A de, Muth D, Westermann F, Speleman F, et al A novel and universal method for microRNA RT-qPCR data normalization. Genome Biol. 2009; 10: R64 doi: 10.1186/gb-2009-10-6-r64 19531210 10.1186/gb-2009-10-6-r64 PMC2718498

[42] Suo C, Salim A, Chia K, Pawitan Y, Calza S. Modified least-variant set normalization for miRNA microarray. RNA. 2010; 16: 2293–2303. doi: 10.1261/rna.2345710 20980676 10.1261/rna.2345710 PMC2995391

[43] Roberts TC, Coenen-Stass AML, Wood MJA, Mukhopadhyay P. Assessment of RT-qPCR Normalization Strategies for Accurate Quantification of Extracellular microRNAs in Murine Serum. PLoS One. 2014; 9: e89237 doi: 10.1371/journal.pone.0089237 24586621 10.1371/journal.pone.0089237 PMC3929707

[44] Sewer A, Gubian S, Kogel U, Veljkovic E, Han W, Hengstermann A, et al Assessment of a novel multi-array normalization method based on spike-in control probes suitable for microRNA datasets with global decreases in expression. BMC Res Notes. 2014; 7: 302 doi: 10.1186/1756-0500-7-302 24886675 10.1186/1756-0500-7-302 PMC4077261

[45] Lv LL, Cao Y, Liu D, Xu M, Liu H, Tang RN, et al Isolation and quantification of microRNAs from urinary exosomes/microvesicles for biomarker discovery. Int J Biol Sci. 2013; 9: 1021–1031. doi: 10.7150/ijbs.6100 24250247 10.7150/ijbs.6100 PMC3831115

[46] Sole C, Cortes-Hernandez J, Felip ML, Vidal M, Ordi-Ros J. miR-29c in urinary exosomes as predictor of early renal fibrosis in lupus nephritis. Nephrol Dial Transplant. 2015; 30: 1488–1496. doi: 10.1093/ndt/gfv128 26040904 10.1093/ndt/gfv128

[47] Ramezani A, Devaney JM, Cohen S, Wing, Scott R, Knoblach S, et al Circulating and urinary microRNA profile in focal segmental glomerulosclerosis: a pilot study. Eur J Clin Invest. 2015; 45: 394–404. doi: 10.1111/eci.12420 25682967 10.1111/eci.12420 PMC4903079

[48] Bryzgunova OE, Zaripov MM, Skvortsova TE, Lekchnov EA, Grigor'eva AE, Zaporozhchenko IA, et al Comparative Study of Extracellular Vesicles from the Urine of Healthy Individuals and Prostate Cancer Patients. PLoS One. 2016; 11: e0157566 doi: 10.1371/journal.pone.0157566 27305142 10.1371/journal.pone.0157566 PMC4909321

[49] Lowery AJ, Miller N, Dwyer RM, Kerin MJ. Dysregulated miR-183 inhibits migration in breast cancer cells. BMC Cancer. 2010; 10: 502 doi: 10.1186/1471-2407-10-502 20858276 10.1186/1471-2407-10-502 PMC2955037

[50] Wang F, Zheng Z, Guo J, Ding X. Correlation and quantitation of microRNA aberrant expression in tissues and sera from patients with breast tumor. Gynecologic Oncology. 2010; 119: 586–593. doi: 10.1016/j.ygyno.2010.07.021 20801493 10.1016/j.ygyno.2010.07.021

[51] Wang J, Chen J, Chang P, LeBlanc A, Li D, Abbruzzesse JL, et al MicroRNAs in Plasma of Pancreatic Ductal Adenocarcinoma Patients as Novel Blood-Based Biomarkers of Disease. Cancer Prevention Research. 2009; 2: 807–813. doi: 10.1158/1940-6207.CAPR-09-0094 19723895 10.1158/1940-6207.CAPR-09-0094 PMC5859193

[52] Wong T, Liu X, Wong BY, Ng RW, Yuen AP, Wei WI. Mature miR-184 as Potential Oncogenic microRNA of Squamous Cell Carcinoma of Tongue. Clin Cancer Res. 2008; 14: 2588–2592. doi: 10.1158/1078-0432.CCR-07-0666 18451220 10.1158/1078-0432.CCR-07-0666

