# Supplementary material for: Identification of miR-16 as an endogenous reference gene for the normalization of urinary exosomal miRNA expression data from CKD patients
Source: PLoS One. 2017 Aug 31;12(8):e0183435. doi: 10.1371/journal.pone.0183435 (PMC5578666; doi:10.1371/journal.pone.0183435)
Supplement: S1 Table — (PDF) [file pone.0183435.s001.pdf]

	miRNA expression normalized against RNA input and IRC			
**Patients/Normal**	**miR-16**	**miR-21**	**miR-92a**	**miR-124a**
1	15.83	15.92	14.91	14.76
2	17.60	18.15	16.49	14.62
3	17.46	17.62	15.47	14.75
4	17.57	18.33	15.67	14.44
5	18.04	18.62	15.84	14.71
6	18.12	18.74	16.29	14.92
7	18.59	18.46	16.05	14.70
8	18.09	18.49	15.75	14.88
9	17.60	18.06	15.88	14.64
10	17.51	18.96	16.16	14.69
11	17.28	17.82	15.64	14.83
12	15.03	16.57	14.40	14.99
13	16.60	17.60	15.80	14.74
14	18.22	19.00	16.57	15.02
15	17.62	18.29	15.80	14.81
16	17.01	18.23	15.06	14.81
17	16.29	18.59	15.17	15.04
18	19.65	17.83	16.29	16.33
19	16.71	15.39	14.37	15.77
20	19.40	17.39	15.88	15.88
21	18.11	16.83	15.39	16.17
22	17.81	16.14	15.14	16.16
23	17.03	15.72	14.38	15.81
24	18.13	16.43	15.52	16.05
25	16.62	15.22	14.77	15.93
26	18.27	15.56	15.28	16.14
27	17.13	15.96	14.62	15.90
28	17.42	16.19	15.04	15.96
29	17.34	16.11	14.98	14.69
30	17.55	17.71	15.94	16.00
31	17.87	16.54	15.27	16.12
32	20.23	20.17	16.07	15.23
33	17.93	16.35	15.04	16.19
N1	16.47	15.93	14.13	16.17
N2	16.70	16.23	14.29	16.12
N3	17.91	16.78	14.86	15.89
N4	17.57	16.68	14.93	15.25
N5	16.88	16.31	14.51	15.01
Average	17.56	17.24	15.36	15.37
